# Cross‐validated permutation feature importance considering correlation between features

**DOI:** 10.1002/ansa.202200018

**Published:** 2022-09-07

**Authors:** Hiromasa Kaneko

**Affiliations:** ^1^ Department of Applied Chemistry, School of Science and Technology Meiji University Kawasaki Japan

**Keywords:** correlation, cross‐validation, feature importance, model interpretation, permutation importance

## Abstract

In molecular design, material design, process design, and process control, it is important not only to construct a model with high predictive ability between explanatory features x and objective features y using a dataset but also to interpret the constructed model. An index of feature importance in x is permutation feature importance (PFI), which can be combined with any regressors and classifiers. However, the PFI becomes unstable when the number of samples is low because it is necessary to divide a dataset into training and validation data when calculating it. Additionally, when there are strongly correlated features in x, the PFI of these features is estimated to be low. Hence, a cross‐validated PFI (CVPFI) method is proposed. CVPFI can be calculated stably, even with a small number of samples, because model construction and feature evaluation are repeated based on cross‐validation. Furthermore, by considering the absolute correlation coefficients between the features, the feature importance can be evaluated appropriately even when there are strongly correlated features in x. Case studies using numerical simulation data and actual compound data showed that the feature importance can be evaluated appropriately using CVPFI compared to PFI. This is possible when the number of samples is low, when linear and nonlinear relationships are mixed between x and y when there are strong correlations between features in x, and when quantised and biased features exist in x. Python codes for CVPFI are available at https://github.com/hkaneko1985/dcekit.

**Abbreviations**
CV: cross‐validation
CVPFI: cross‐validated permutation feature importance
DT: decision tree
GMR: Gaussian mixture regression
GPR: Gaussian process regression
LIME: local interpretable model‐agnostic explanations
PFI: permutation feature importance
PLS: partial least squares
RF: random forests
SHAP: Shapley additive explanations
SVR: support vector regression
VD: validation data

## 1 INTRODUCTION

In molecular design, material design, process design, process control, and process management, it is common to utilise mathematical models y = f(x) constructed between objective features y and explanatory features x using a dataset. An important objective is to construct models with high prediction accuracy. Classification and regression methods include linear discriminant analysis, logistic regression,^1^ partial least squares (PLS) regression,^2^ ridge regression, least absolute shrinkage and selection operator, elastic net,^3^ support vector regression (SVR),^1^ decision tree,^4^ random forests (RF),^5^ Gaussian process regression (GPR),^1^ gradient boosting decision tree,^6^ extreme gradient boosting,^7^ light gradient boosting machine,^8^, ^9^, ^10^ CatBoost,^11^, ^12^ deep neural network,^13^ and Gaussian mixture regression (GMR).^14^, ^15^ Since there exists no optimal classification method or optimal regression method, a method that is appropriate for each dataset should be used.

It is also important to interpret the constructed models and analyse the relationship between x and y to elucidate the mechanism by which the physical properties and activities are expressed. In local interpretable model‐agnostic explanations (LIME)^16^ and Shapley additive explanations (SHAP),^17^ which can be combined with any regression method, the slope of x with respect to y around a sample point is determined by obtaining an approximate expression for the shape of the model at the sample point. LIME and SHAP can be used to discuss the local contribution or direction of x to y. For example, for a sample with a maximum value of y, we can discuss the direction of x to further improve the y value. However, feature importance, which is the degree of influence of each x on y, in the entire dataset is the focus of this study.

Several methods exist for establishing the feature importance of RF models^18^, ^19^, ^20^, ^21^, ^22^, ^23^, ^24^, ^25^, ^26^ such as mean decrease impurity and permutation feature importance (PFI). The feature importance of x is calculated considering the entire value of y but it can be different when the y value is high, medium, or low. Shimizu and Kaneko (2021) proposed a decision tree (DT) and RF hybrid model where the importance of RF was calculated for each leaf node in the DT model, and thus, DT provided a global interpretation of the entire dataset, and RF provided local interpretations for each cluster.^27^

PFI can be used universally with various classification and regression methods such as scikit‐learn^28^ and also, can be used conveniently. However, validation data (VD) and training data are required to calculate the PFI, and the feature importance calculation is unstable when the number of samples is low. Additionally, the PFI of strongly correlated features is estimated to be lower than that of other independent features.

Therefore, in this study, cross‐validated permutation feature importance (CVPFI) is proposed to solve the above problems and calculate the feature importance appropriately. Because CVPFI is calculated in an iterative manner based on cross‐validation (CV), the feature importance can be calculated stably by increasing the number of divisions for CV when the number of samples is low. Additionally, when randomly shuffling a feature, it is possible to estimate the importance of strongly correlated features appropriately by shuffling other correlated features with probability based on the absolute correlation coefficients between the features.

The performance of CVPFI is verified using numerical simulation data generated in cases where x and y are linearly related to small samples, where x and y are nonlinearly related, where highly correlated features exist in x, and where quantised features whose values are unbalanced exist in x, compared with PFI. The CVPFI of each descriptor was then discussed using a dataset of actual compounds.

The paper is organised as follows. Section 2 highlights the description of the methods used in the paper. Section 3 presents the results of the empirical analysis and discusses the results. Lastly, Section 4 concludes the study and provides implications of the work.

## 2 METHOD

### 2.1 Permutation feature importance

When the number of iterations is *J*, the algorithm for calculating the PFI is as follows:

1. Construct a model using training data.
2. Calculate the reference score *rs* of the model on VD. The score is the accuracy of the classifier and the determination coefficient r^2^ for a regressor.
3. For each feature *i*, which means the *i*th column of VD, and for each repetition *j* in 1, 2,…, and *J*, randomly shuffle column *i* or *i*th feature of dataset *VD* to generate a corrupted version of VD CVD*i*,*j* and calculate the score *s_i,j_* of the model on CVD*i*,*j*.
4. Calculate the importance of *PFI_i_* for the *i*th feature as follows:
(1) $$PFI_{i}=rs-\frac{1}{J}\sum_{j=1}^{J}s_{i,j}$$

### 2.2 Cross‐validated permutation feature importance

**FIGURE 1:**
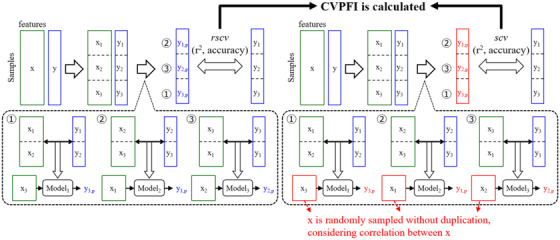
Basic concept of cross‐validated permutation feature importance

Figure 1 shows the basic concept of the CVPFI. In PFI, the score *s_i_*_,_ *_j_* was calculated using a single set of VD; however, in CVPFI, the score was calculated using CV. For CV, because all samples become VD after the iterative calculation, the feature importance will be calculated stably even when the number of samples is low. However, when predicting VD in CV, if the *i*th feature of VD is randomly shuffled, as is the case with PFI, the feature will not be shuffled effectively when the number of divisions or folds of the CV is large. For example, in leave‐one‐out CV, the *i*th feature cannot be shuffled because there exists only one sample in VD per fold. Therefore, in CVPFI, instead of shuffling the *i*th feature of VD only, the *i*th feature of VD is randomly sampled without duplication from the original dataset. The proposed method increases the number of samples for model construction and model evaluation compared to the conventional method; thus, feature importance can be calculated stably using the proposed method.

Additionally, only the *i*th feature is shuffled when calculating *s_i_*_,_ *_j_* in PFI; however, in CVPFI, not only the *i*th feature but also other features correlated with the *i*th feature can be randomly sampled without duplication from the original dataset. In general, when there exists a correlation between two features and one of them changes, the other one also changes according to its correlation, which is replicated in CVPFI. The higher the correlation with the *i*th feature, the higher the probability of being randomly sampled. Thus, the probability was set as the absolute value of the correlation coefficient with the *i*th feature in the CVPFI. However, it is necessary to consider chance correlation, particularly when the number of samples is low. When the absolute value of the correlation coefficient is higher than zero and there is essentially no correlation between the features, it becomes noise, leading to false feature importance. Therefore, interval estimation of the population correlation coefficient^29^ is performed before the probability is set as the absolute correlation coefficient. When the estimated interval includes zero, that is, when the product of the lower and upper limits of the correlation coefficient is less than zero, the correlation coefficient is set to zero, that is, there is no correlation.

In the interval estimation of the population correlation coefficient, the absolute correlation coefficient between *p*th and *q*th features, *r_p_*_,_ *_q_* is converted to *z_p_*_,_ *_q_* by Fisher z‐transformation^29^ using the following equation:

(2) $$z_{p,q}=\frac{1}{2}\log\frac{1+r_{p,q}}{1-r_{p,q}}$$

Because it can be assumed that z follows a normal distribution with a mean of *z_p_*_,_ *_q_* and a variance of 1/(*m*−3), the range of z whose probability is *α* (the significance level) can be estimated. The lower and upper limits of this range are denoted by *Lz_p_*_,_ *_q_* and *Uz_p_*_,_ *_q_*, respectively. In this study, scipy.norm.interval^30^ was used to calculate them.

*Lz_p_*_,_ *_q_* and *Uz_p_*_,_ *_q_* of z are converted to *Lr_p_*_,_ *_q_* and *Ur_p_*_,_ *_q_* of r, respectively, as follows:

(3) $$Lr_{p,q}=\frac{\exp\left(2Lz_{p,q}\right)-1}{\exp\left(2Lz_{p,q}\right)+1}$$

(4) $$Ur_{p,q}=\frac{\exp\left(2Uz_{p,q}\right)-1}{\exp\left(2Uz_{p,q}\right)+1}$$

When *Lr_p_*_,_ *_q_* < 0 < *Ur_p_*_,_ *_q_*, the correlation has the possibility of chance correlation; thus, *r_p_*_,_ *_q_* = 0. When feature A is important and feature B is correlated with A, then B is also important, at least by the amount of its correlation coefficient with A. Of course, only correlations between features cannot extract all relationships among features in a multivariate dataset; however, the correlation coefficients can represent some necessary relationships between features, and the CVPFI is calculated by considering the relationships.

When the number of iterations is *J*, the algorithm for calculating CVPFI is given as follows:

1. Calculate the correlation coefficients between all the features.
2. Calculate *Lr_p_*_,_ *_q_* and *Ur_p_*_,_ *_q_* between all features and *r_p_*_,_ *_q_* = 0 when *Lr_p_*_,_ *_q_* < 0 < *Ur_p_*_,_ *_q_*.
3. In CV, for *n* = 1, 2, …, *N*, where *N* is the number of folds of CV, the following procedures are conducted using training data and VD at each fold.
  3‐1 Construct a model using the *n*th training data.
  3‐2 Estimate y values on *n*th VD VD*_n_* using the model
  3‐3 For each feature *i*, which is the *i*th column of VD*_n_*, and for each repetition *j* in 1, 2,…, *J*, randomly sample column *i* of the original dataset without duplication, and for each feature *m* (the *m*th column of VD*_n_*) for which *r_i_*_,_ *_m_* is higher than 0, randomly sample column *m* of the original dataset without duplication with a probability of *r_i_*_,_ *_m_* to generate a corrupted version of the dataset CVD*_n,i_*_,_ *_j_*, and estimate the y values of the model.
4. Integrate the y‐values estimated in CV for VD_1_, VD_2_ *,…*, and VD*_N_*, and calculate the reference score *rscv* with the integrated y‐values. The score is the accuracy of the classifier and the determination coefficient *r* ^2^ for a regressor.
5. Integrate the y‐values estimated in CV for CVD_1_ *_,i_*_,_ *_j_*, CVD_2_ *_,i_*_,_ *_j,_,…*, and CVD*_N,i_*_,_ *_j_*, and calculate the score *scv_i,j_* with the integrated y‐values.
6. Calculate the importance of *CVPFI_i_* for the *i*th feature as follows:
(5) $$CVPFI_{i}=rscv-\frac{1}{J}\sum_{j=1}^{J}scv_{i,j}$$

Because CVPFI integrates estimated y‐values and the number of samples to calculate the accuracy for a classifier, and r^2^ for a regressor is larger than that of PFI, the importance can be calculated stably in CVPFI.

Python code for CVPFI is available at https://github.com/hkaneko1985/dcekit. In this code, the maximal information coefficient^31^ can be used instead of the correlation coefficient r.

## 3 RESULTS AND DISCUSSION

To validate the proposed CVPFI, it was compared with the conventional PFI using numerical simulation data and actual compound data. *J* was set equal to 5 for both the PFI and CVPFI. For PFI, the data were randomly split so that the training data and validation data contained 75% and 25% of the samples, respectively. For CVPFI, *α* was set to 0.999. A leave‐one‐out CV was used when the number of samples was less than 30, a 10‐fold CV when the number of samples was higher than 30 but less than 100, and a 5‐fold CV when the number of samples was higher than 100. In this paper, PLS, which is a linear regression method, SVR with Gaussian kernel, and GPR, which are nonlinear regression methods, and GMR, which is a nonlinear regression method enabling direct inverse analysis, were used. The kernel function used in the GPR is given as follows:

(6) $$K\left(x^{\left(i\right)},x^{\left(j\right)}\right)=\theta_{0}\exp\left\{-\frac{\theta_{1}}{2}{\left\|x^{\left(i\right)}-x^{\left(j\right)}\right\|}^{2}\right\}+\theta_{2}$$

where **x** ^(^ *^i^* ^)^ is the *i*th sample of x and *θ*_0_, *θ*_1_, and *θ*_2_ are the hyperparameters.

In numerical simulation data, the following four case studies were conducted:

- Case study 1: There are linear relationships between 10 features in x and y, five features unrelated to y are included in x, and the number of samples is low.
- Case study 2: There are linear and non‐linear relationships between 10 features in x and y, five features unrelated to y are included in x, and the number of samples is low.
- Case study 3: There is a linear relationship between x and y, and x contains features that are strongly correlated.
- Case study 4: There is a linear relationship between x and y, and x contains quantised features with biased values.

In case study 1, x was set to 15, and samples of x were generated as uniform random numbers between 0 and 1. The first 10 features in x had weights to y, and all the weights were one. The remaining five features in x have no contribution to y, as do all the weights of zero. Normal random numbers with a standard deviation of 10% were added to y. The sample size was set at 20.

**FIGURE 2:**
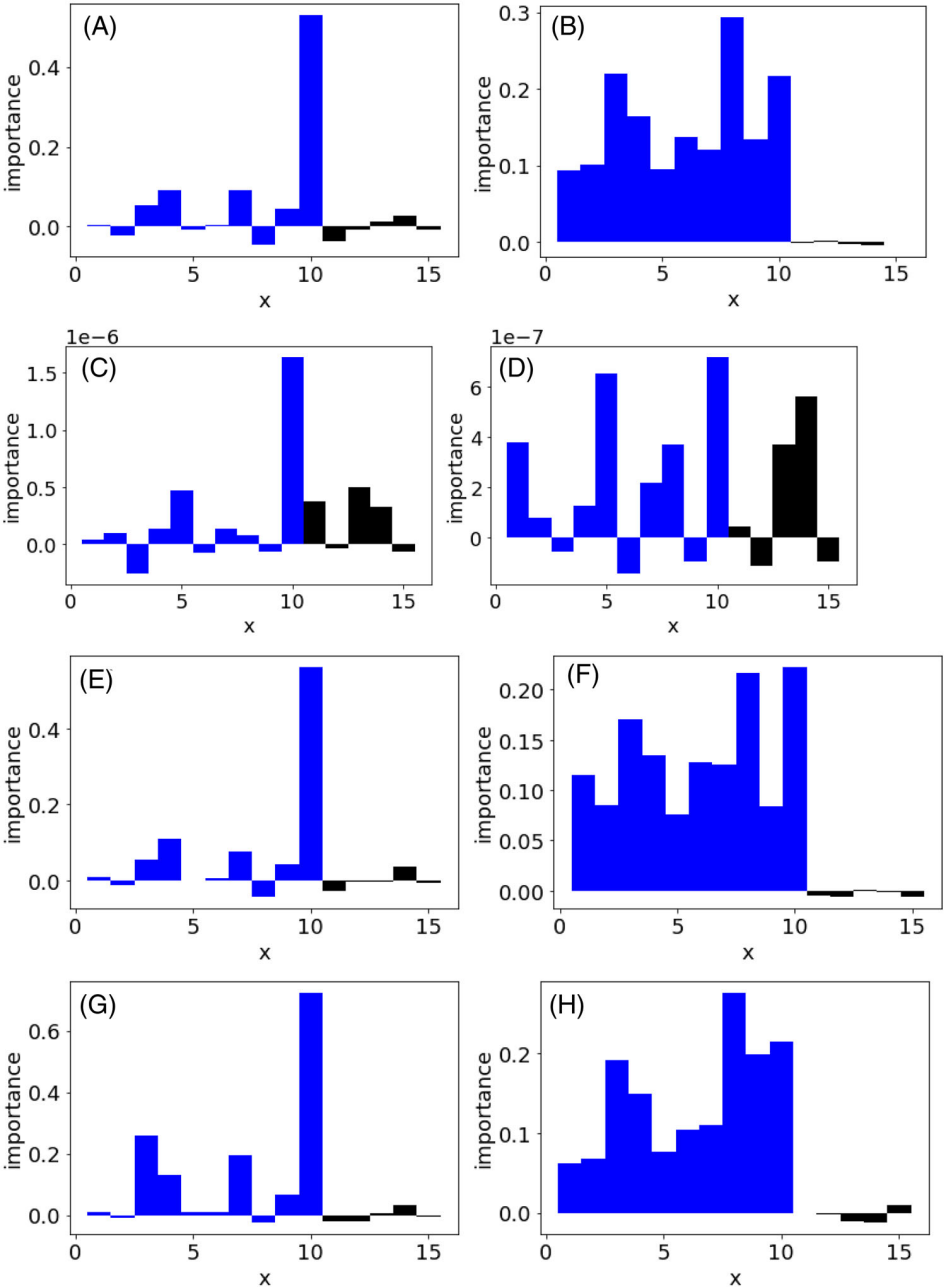
Feature importance in case study 1. Blue and black bars correspond to significant x and non‐significant x, respectively. (A) PFI in PLS, (B) CVPFI in PLS, (C) PFI in SVR, (D) CVPFI in SVR, (E) PFI in GPR, (F) CVPFI in GPR, (G) PFI in GMR and (H) CVPFI in GMR. Note: PFI, permutation feature importance; PLS, partial least squares; CVPFI, cross‐validated permutation feature importance; SVR, support vector regression; GPR, Gaussian process regression; GMR, Gaussian mixture regression

Figure 2 shows the feature importance of each regression method when the PFI and CVPFI are used in case study 1. The results of x of the first to 10th features related to y are shown as blue bars, and the results of x of the 11th to 10th features not related to y are shown as black bars. The PFI results in Figure 2A,C,E,G shows that in the first to 10th features, some features have high importance, but the importance of several features is lower than the maximum importance of the features not related to y. However, Figure 2B,D,F,H shows that the importance of the features related to y is sufficiently high compared to the maximum importance of the features not related to y in all results except the SVR result. Because the number of samples was low and PFI was unstable, the importance of features not related to y was higher than that of features related to y, although the importance of the 10th feature was high. However, the proposed CVPFI could evaluate feature importance appropriately, and the importance of features unrelated to y was lower than that of features related to y. The proposed method can properly evaluate the important features. In SVR, because the number of samples was small and there were three hyperparameters, the optimisation of the hyperparameters in SVR would fail, and SVR could not model the relationship between x and y on the present dataset. It was found that the feature importance could be appropriately evaluated even in a small number of samples using the proposed CVPFI.

In case study 2, x was set to 15, and samples of x were generated as uniform random numbers between 0 and 1. The first five features in x contribute to y as follows:

(7) $$\begin{array}{ccc} y & = & {\left(3x_{1}-1.5\right)}^{2}+{\left(2x_{2}-1\right)}^{3}+\exp\left(x_{3}-0.5\right)+2\log\left(x_{4}+1\right)\\ & & +\;1.5\sin\left(x_{5}\right) \end{array}$$

The next five features in x had weights of y, and all the weights were one. The remaining five features in x have no contribution to y, as do all the weights of zero. Normal random numbers with a standard deviation of 10% were added to y. The sample size was set to 60.

**FIGURE 3:**
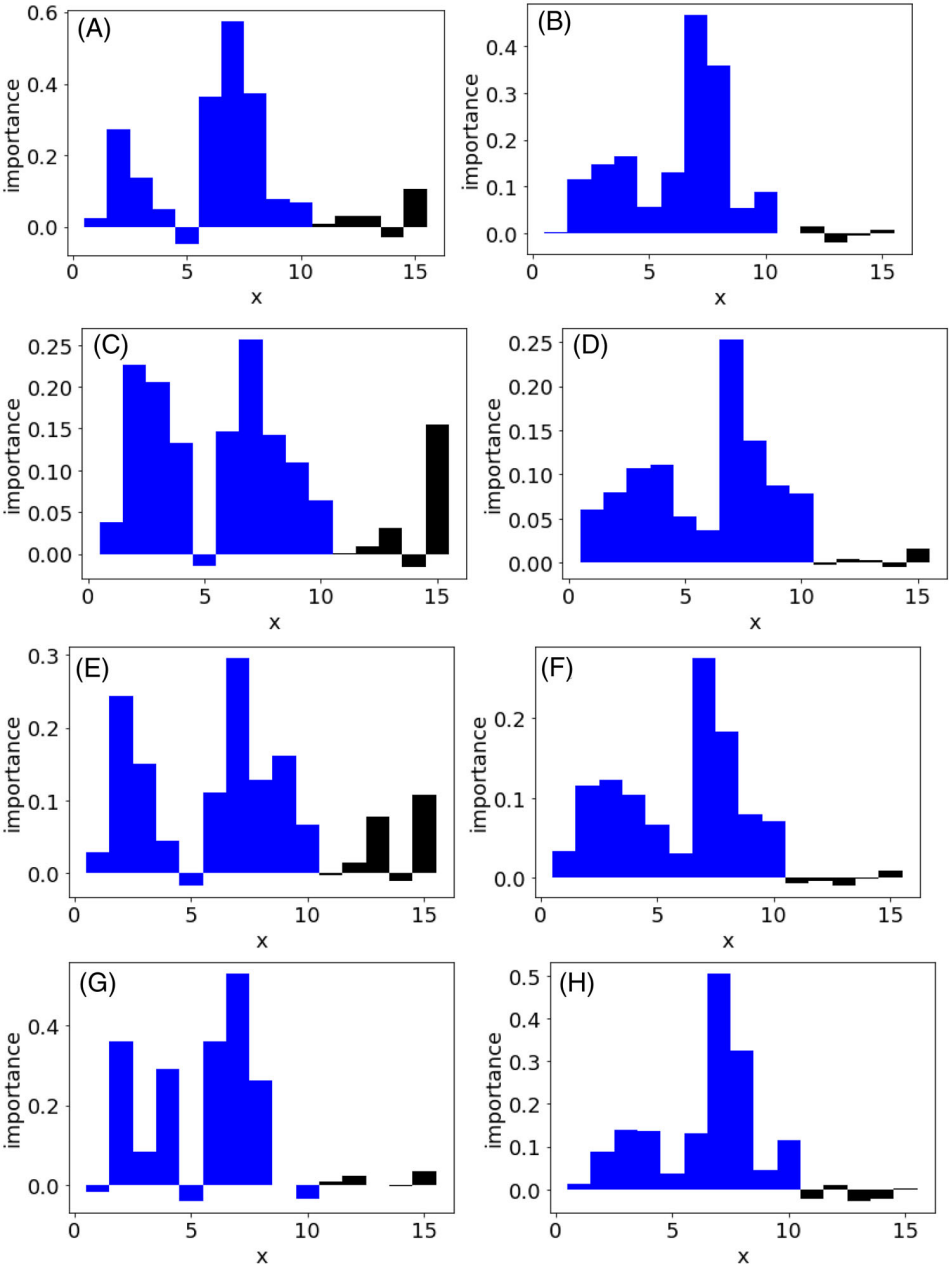
Feature importance in case study 2. Blue and black bars correspond to significant x and non‐significant x, respectively. (A) PFI in PLS, (B) CVPFI in PLS, (C) PFI in SVR, (D) CVPFI in SVR, (E) PFI in GPR, (F) CVPFI in GPR, (G) PFI in GMR and (H) CVPFI in GMR. Note: PFI, permutation feature importance; PLS, partial least squares; CVPFI, cross‐validated permutation feature importance; SVR, support vector regression; GPR, Gaussian process regression; GMR, Gaussian mixture regression

Figure 3 shows the feature importance of each regression method while using the PFI and CVPFI in case study 2. The results of x of the first to 10th features related to y are shown as blue bars, and the results of x of the 11th to 10th features not related to y are shown as black bars. The PFI results in Figure 3A,C,E,G shows that the number of important features whose importance exceeds the importance of the 15th feature unrelated to y is low, and many important features in x are considered less important than the unimportant feature. However, Figure 3B,D,F,H shows that the importance of the features related to y is higher than the maximum importance of the features unrelated to y for all the nonlinear regression methods, that is, SVR, GPR, and GMR. In the linear PLS, although the feature importance of x_1_ with a nonlinear relationship to y was low, the importance of the other important features in x was appropriately high. It was found that the combination of the proposed CVPFI and nonlinear regression method can properly evaluate the feature importance even when there are linear and nonlinear relationships between x and y with small data.

In case study 3, the number of x was set to 10, and the first five features were generated as uniform random numbers between 0 and 1. and Then, normal random numbers with a standard deviation of 10% were added for each feature. These features are independent of one another. For the five features to be highly correlated with each other, the *i*th x, x*_i_*, is generated as follows:

(8) $$x_{i}=u+0.1\times N\left(0,1\right).$$

Here, u is a uniform random number between 0 and 1 and *N*(0, 1) is the standard normal random number. After standardising all 10 features, all the weights to y were set to one, and then the contributions of features for y were equivalent for all the features. Normal random numbers with a standard deviation of 10% were added to y. The sample size used was 100 µm.

**FIGURE 4:**
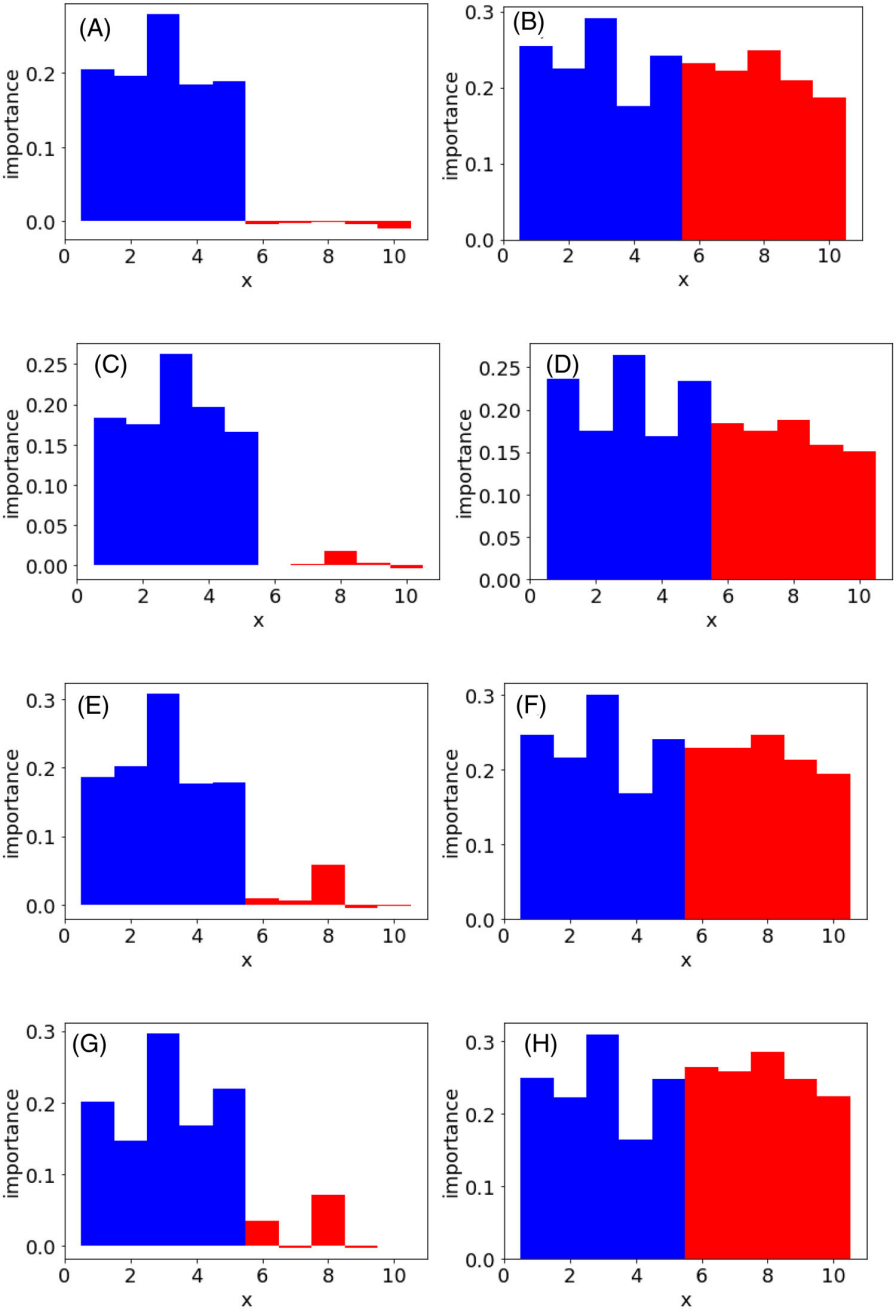
Feature importance in case study 3. Blue and red bars correspond to independent x and strongly correlated x, respectively. (A) PFI in PLS, (B) CVPFI in PLS, (C) PFI in SVR, (D) CVPFI in SVR, (E) PFI in GPR, (F) CVPFI in GPR, (G) PFI in GMR and (H) CVPFI in GMR. Note: PFI, permutation feature importance; PLS, partial least squares; CVPFI, cross‐validated permutation feature importance; SVR, support vector regression; GPR, Gaussian process regression; GMR, Gaussian mixture regression

Figure 4 shows the feature importance of each regression method when the PFI and CVPFI are used in case study 3. The results of x for the first to fifth features uncorrelated with each other are shown as blue bars, and the results of x for the sixth to 10th features highly correlated with each other are shown as red bars. From the PFI results in Figure 4A,C,E,G, the importance of the features that are highly correlated with each other is much lower than the importance of the features that are uncorrelated with each other in all the regression methods, although the weights to y are the same for all the features in x. This could be because PFI did not consider correlation in x at all, and other correlated x could explain y in permutation. However, Figures 4B and 3D,F,H show that by using CVPFI and considering the correlation between features in permutation, it did not matter whether the features were uncorrelated or strongly correlated with each other, and the importance of features could be evaluated at the same level of importance in all the regression methods. It was confirmed that feature importance can be evaluated in the same way as independent features, even when features that are highly correlated with each other are included in x using the proposed CVPFI.

In case study 4, x was set to 10, and the first five features were generated as uniform random numbers ranging from 0 to 1. The remaining five features were generated to have only 0 or 1 and to be biased such that the percentage of 1 was 10%. After standardising all features, all weights to y were set to 1, and y was calculated. Normal random numbers with a standard deviation of 10% were added to y. The sample size used was 100 µm.

**FIGURE 5:**
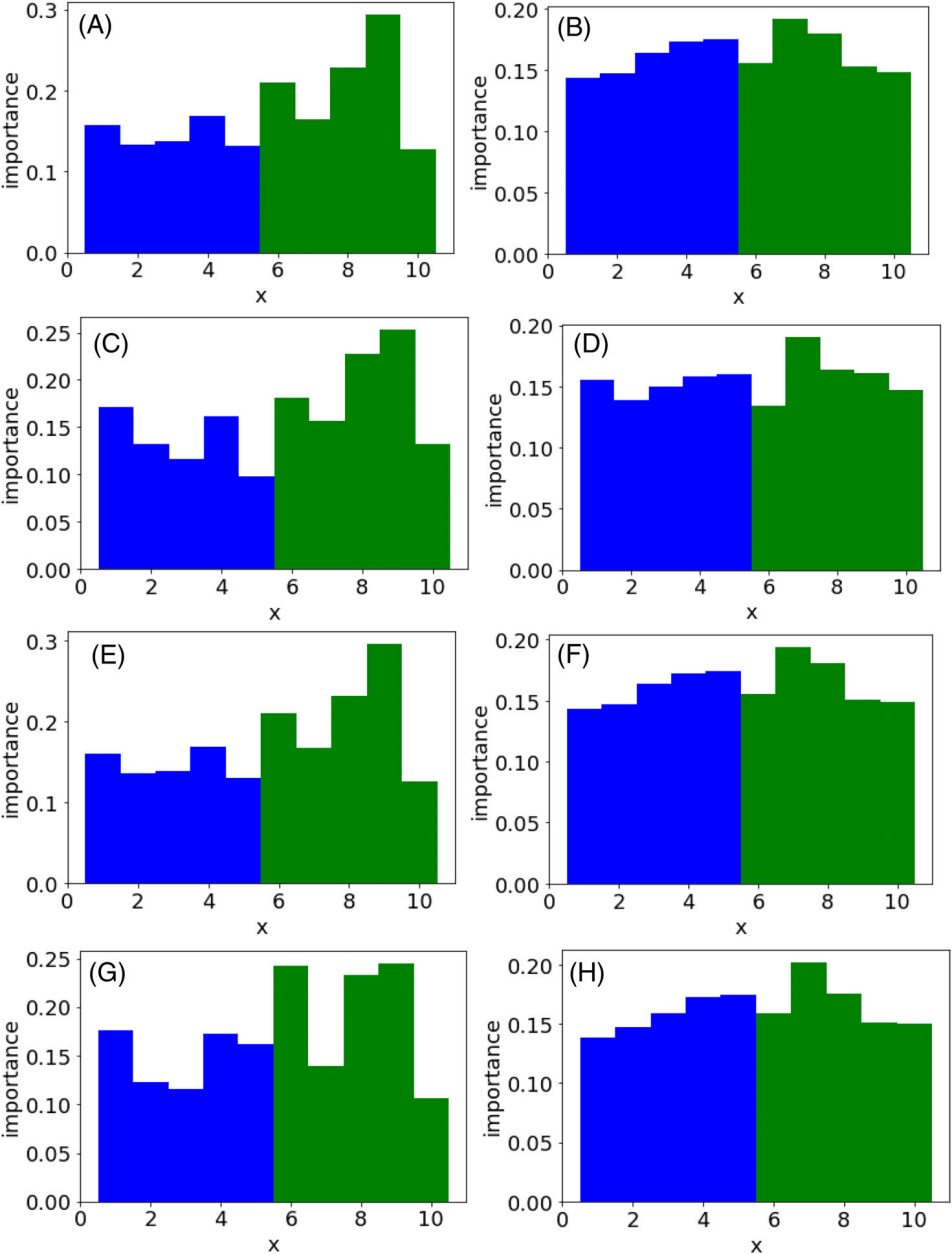
Feature importance in case study 4. Blue and green bars correspond to continuous x and quantised x, respectively. (A) PFI in PLS, (B) CVPFI in PLS, (C) PFI in SVR, (D) CVPFI in SVR, (E) PFI in GPR, (F) CVPFI in GPR, (G) PFI in GMR and (H) CVPFI in GMR. Note: PFI, permutation feature importance; PLS, partial least squares; CVPFI, cross‐validated permutation feature importance; SVR, support vector regression; GPR, Gaussian process regression; GMR, Gaussian mixture regression

Figure 5 shows the feature importance of each regression method when using the PFI and CVPFI in case study 4. The results of x for the first to fifth continuous features are shown as blue bars, and the results of x for the sixth to 10th quantised features with biased values are shown as green bars. For both PFI and CVPFI, feature importance can be evaluated as having the same level of importance, whether they are continuous or quantised features. In terms of the variation of importance between features, that of CVPFI was lower than that of PFI, indicating that CVPFI was more stable than PFI because the dataset in case study 4 was generated so that the weights of x to y were the same. It was found that the proposed CVPFI can stably evaluate feature importance even when quantised features with biased values exist.

Next, to examine the performance of the proposed method, a dataset of boiling points^32^ was used as the actual dataset for the compounds. RDKit^33^ was used to calculate the molecular descriptors, and only interpretable descriptors were selected to test the interpretability of the model. Those features for which the ratio of samples with the same values in the training data accounted for 80% or more were excluded. One of the pairs of features for which the absolute correlation coefficient attained a value of one was subsequently deleted. The descriptors used in this study are listed in Table 1.

**TABLE 1:** Molecular descriptors of RDKit used in this study

Name	Description
MolWt	Average molecular weight of the molecule
HeavyAtomMolWt	Average molecular weight of the molecule ignoring hydrogens
ExactMolWt	Exact molecular weight of the molecule
NumValenceElectrons	Number of valence electrons that the molecule has
HeavyAtomCount	Number of heavy atoms a molecule
NHOHCount	Number of NHs or OHs
NOCount	Number of nitrogens and oxygens
NumAromaticRings	Number of aromatic rings
NumHAcceptors	Number of hydrogen bond acceptors
NumHDonors	Number of hydrogen bond donors
NumHeteroatoms	Number of heteroatoms
NumRotatableBonds	Number of rotatable bonds
RingCount	Number of rings
MolLogP	Wildman‐Crippen LogP value
MolMR	Wildman‐Crippen MR value
fr_C_O	Number of carbonyl O
fr_C_O_noCOO	Number of carbonyl O, excluding COOH

**FIGURE 6:**
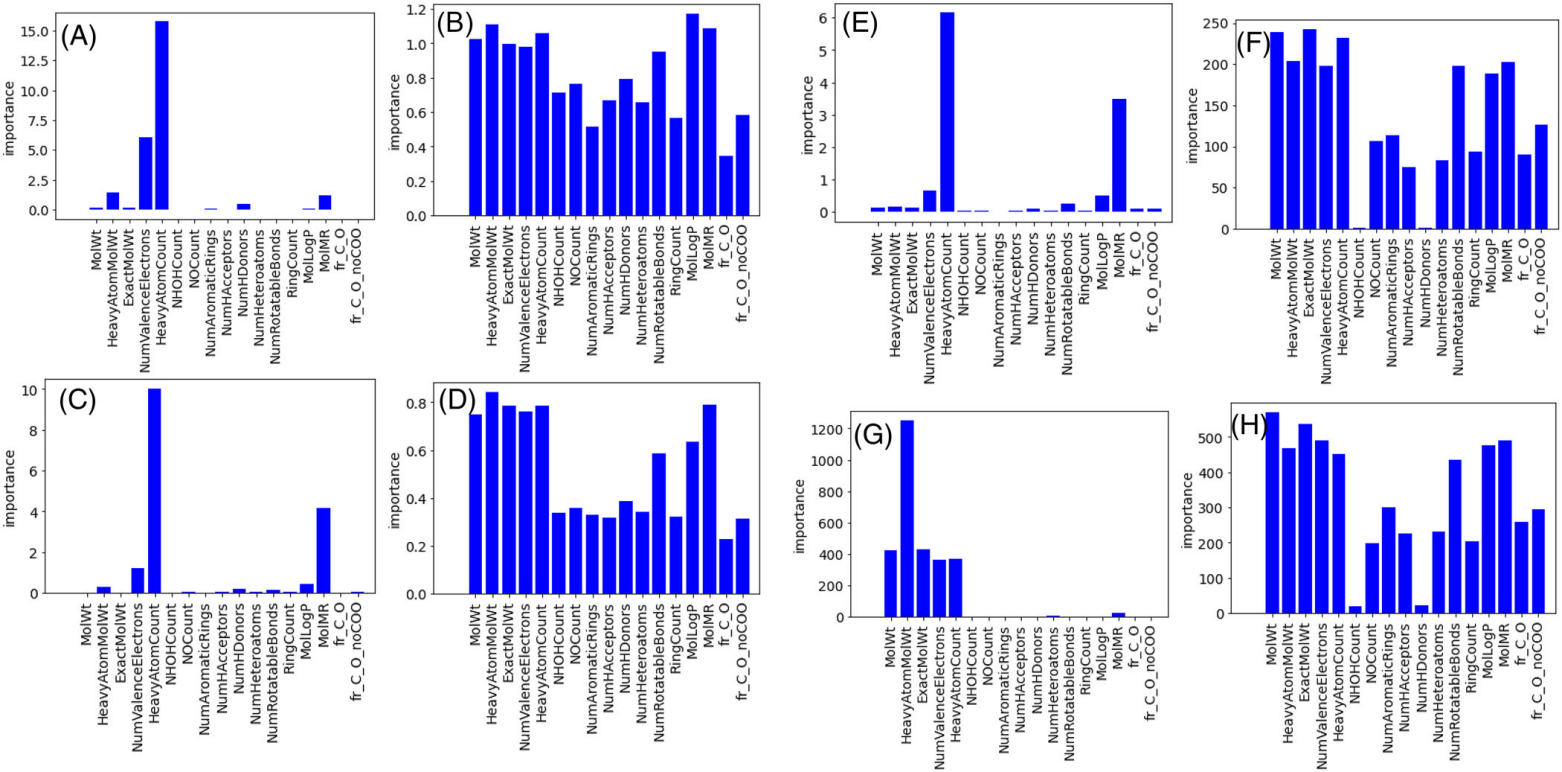
Feature importance for the boiling point dataset. (A) PFI in PLS, (B) CVPFI in PLS, (C) PFI in SVR, (D) CVPFI in SVR, (E) PFI in GPR, (F) CVPFI in GPR, (G) PFI in GMR and (H) CVPFI in GMR. Note: PFI, permutation feature importance; PLS, partial least squares; CVPFI, cross‐validated permutation feature importance; SVR, support vector regression; GPR, Gaussian process regression; GMR, Gaussian mixture regression

Figure 6 illustrates the feature importance of each regression method when PFI and CVPFI were used in the boiling point dataset. Although molecular weight is one of the important factors affecting the boiling point, the PFI results (Figure 6A,C,E,G) show that the importance of all features related to molecular weight is low, except for GMR. Additionally, the features related to the number of atoms and substructures are also important in explaining the boiling point; however, they became less important for all regression methods. The features, including molecular weight and the number of atoms and substructures, are considered less important owing to the correlation between the features. However, Figure 6B,D,F,H shows that by using CVPFI, the importance of the features related to molecular weight and the number of atoms and substructure increased, and the results were reasonable for all regression methods. It was found that the proposed CVPFI can be used to properly evaluate the importance of features in a real dataset where correlations exist between the features.

## 4 CONCLUSION

In this study, the CVPFI was proposed to properly evaluate feature importance using the machine learning method. Compared with the conventional PFI, CVPFI can calculate the feature importance stably and appropriately because the model construction and evaluation of features are repeated based on CV. Furthermore, since features other than the target feature of permutation are randomly sampled based on the correlation coefficients of the features, the importance of strongly correlated features can be evaluated appropriately as well as independent features.

Through case studies using numerical simulation data, it was confirmed that CVPFI can be used to evaluate the feature importance appropriately compared to the conventional PFI in all cases where the number of samples is low, where linear and nonlinear relationships are mixed between x and y, where features with strong correlation exist in x, and where features that are quantised and have biased values exist in x. Furthermore, when the actual boiling point dataset was used, the feature importance could be properly evaluated in the presence of correlations between molecular descriptors. Although CVPFI was applied only to regression analysis in this study, it can also be used for classification by changing the evaluation index of the model from r^2^ to an index of classification, such as accuracy and Cohen's kappa.

Although the CVPFI can be combined with any regression method, the number of years that can be considered simultaneously depends on the regression method. For example, when using SVR, only one y can be considered; however, when using GMR, any y can be considered simultaneously. The proposed CVPFI is expected to facilitate the interpretation of data‐driven models, explanation of phenomena, and clarification of mechanisms in datasets.

Python codes for CVPFI are available.^34^

## CONFLICT OF INTEREST

The authors declare there is no conflict of interest.

## Data Availability

The data that support the findings of this study are available in reference number [32].
